# Serum Lactate Is not Correlated with Mixed or Central Venous Oxygen Saturation for Detecting Tissue Hypo Perfusion During Coronary Artery Bypass Graft Surgery: A Prospective Observational Study

**Published:** 2013-12-01

**Authors:** Shahrbano Shahbazi, Saeed Khademi, Masih Shafa, Reza Joybar, Maryam Hadibarhaghtalab, Mohammad Ali Sahmeddini

**Affiliations:** 1 Shiraz Anesthesiology and Intensive Care Research Center, Shiraz University of Medical Sciences, Shiraz, IR Iran; 2 Department of Cardiac Surgery, Shiraz University of Medical Sciences, Shiraz, IR Iran; 3 Student Research Committee, Fasa University of Medical Sciences, Fasa, IR Iran

**Keywords:** Lactates, Coronary Artery Bypass, Catheterization

## Abstract

**Objectives:**

Effective assessment of tissue perfusion is highly important during Coronary Artery Bypass Graft (CABG). Mixed venous O_2_ saturation (Svo_2_) is one of the best and routinely used markers of tissue perfusion. However, this method is costly and leads to considerable complications. Thus, the present study aimed to determine whether the Svo_2_ can be substituted with central venous saturation (Scvo_2_) and if there is any correlation between lactate level and Svo_2_.

**Methods:**

This prospective observational study was conducted on 62 patients scheduled for CABG. After induction and maintenance of anesthesia, blood samples drawn from central venous, pulmonary artery, and radial artery were used to measure Scvo_2_, Svo_2_ and serum lactate level respectively before and after Cardio Pulmonary Bypass (CPB). Pearson’s correlation test was used to determine the correlation between Svo_2_ and Scvo_2_ as well as between Svo_2_ and serum lactate level. Besides, P < 0.05 was considered as statistically significant.

**Results:**

Overall, 62 Patients, 33 males (53.2%) and 29 females (46.8%) were enrolled into the present study. The most common coexisting illness was hypertension detected in 33 patients (53.2%) followed by hypercholesterolemia in 28 ones (44.4%). In this study, Svo_2_ was positively correlated with Scvo_2_ (r = 0.63, P < 0.001). However, no correlation was found between Svo2 and lactate (r = 0.124, P = 0.348).

**Conclusions:**

In summary, Scvo_2_ is considered as the best substitute of Svo_2_ for detecting tissue hypo perfusion during CPB. Although the lactate level had been considered as an appropriate marker of tissue perfusion and ischemia, it was not correlated to Svo_2_ during CABG.

## 1. Background

Detection of tissue hypoperfusion is a serious concern for cardiac anesthesiologist during Coronary Artery Bypass Graft (CABG). This is due to the fact that it is not only associated with high in-hospital mortality, but also aggressive therapy to restore tissue perfusion could substantially reduce mortality (1). Tissue hypoperfusion is usually detected by decrease in mixed venous oxygen saturation or increase in serum lactate levels (2). Perz et al. also proposed that the combined analysis of Svo_2_ and lactate level might be used for early detection of the patients at risk of tissue hypoperfusion (3). Measurement of Svo_2_ as the best marker of tissue perfusion is very difficult since it is done through correctly positioning the Pulmonary Artery Catheter (PAC). In addition, this method is invasive, costly, and may lead to considerable complications during and after the surgery (4).

Yet, there are other methods which are less invasive for detecting tissue hypoperfusion (5). One of these methods is measurement of serum lactate, during CPB. Hyperlactatemia appears to be mainly due to insufficient oxygen delivery (6-8). Although some studies have shown an association between serum lactate levels and severity of the complications after the CABG (9, 10), others argue that this is not a sensitive marker (5, 11, 12). The second marker for detecting tissue hypoperfusion is measurement of central venous oxygen saturation (Scvo_2_) which is done by using a Central Venous Catheter (CVC). This method is slightly less invasive and less costly compared to PAC. Even though some studies have proved that Scvo_2_ is sufficiently parallel to Svo_2_ or is appropriate monitoring especially if Scvo_2_ is continuously monitored (13, 14), others have mentioned that Svo_2_ could not be replaced by Scvo_2_ in the patients undergoing cardiac surgery (1).

To date, there has not any report available regarding the possible relationship between serum lactate level and Svo_2_ during the CABG surgery. In addition, it is still unclear whether Svo_2_ and Scvo_2_ can be substituted. Thus, the present study is conducted to investigate the possibility of substitution of the traditional marker of tissue hypo perfusion (Svo_2_) with new markers of tissue hypoperfusion, such as Scvo_2_ and serum lactate level.2.

## 2. Patients and Methods

This prospective observational study was conducted in the department of cardiac surgery in Nemazee hospital, Shiraz, Iran. With the approval of our Institutional Ethics Committee and obtaining written informed consents, 62 patients who had been scheduled for elective CABG from November 2010 to February 2011 were enrolled into the study. The patients were excluded if they had a history of severe hepatic, renal dysfunction and Chronic Obstructive Pulmonary Disease (COPD) with Forced Expiratory Volume in 1 second (FEV1) < 50%. The patients undergoing combined surgical procedures (CABG combined with valve surgery or aortic procedures and carotid surgery) and redo procedure, those under 25 years of age, those with Body Mass Index (BMI) > 32 kg / m2, pregnant women, the patients with sepsis, and those in the shock state were excluded from the study, as well.

All the patients were premedicated with Oxazepam 10 mg PO before the surgery. Peripheral venous access and then radial artery cannulae were inserted before the induction of anaesthesia. Anaesthesia was induced with midazolam 0.1 mg / kg, sufentanil 0.75 - 1 μg / kg, morphine 0.1 mg / kg, pancuronium 0.1 - 0.15 mg / kg, and thiopental 1 - 2 mg / kg. In addition, anaesthesia was maintained with Isoflurane 0.5 - 1.5% and all the patients were ventilated with an oxygen-air mixture (50% - 50%) to maintain an end-tidal CO_2_ of 35 to 45 mmHg. Also, all the patients underwent nonpulsatile, normothermic (34 - 36°C) CPB with intermittent cold blood cardioplegia for cardiac quiescence. The extracorporeal circuit was primed with 1000 - 1500 mL of Ringer's solution and 250 - 500 mL of 6% voluven solution. Serial hematocrits (Hct) were kept above 18 with packed red blood cell transfusion as necessary during CPB. Besides, the mean blood pressure was maintained between 60 and 90 mmHg with pump flow rate of 2 to 2.4 L / min / m2 (by stoker S5, 2010 Germany) throughout CPB.

Immediately after induction of anesthesia and tracheal intubation, central venous catheter (CVC) – a triple lumen spectrophotometer catheter – was inserted in the right internal jugular vein and adjusted to obtain a sample for measuring the central venous oxygen saturation (Scvo_2_) before the initiation (T1) and after the termination of cardiopulmonary bypass (CPB) (T2). The blood samples collected from arterial catheter were used to measure the lactate level before the initiation (T1) and after the termination of CPB (T2).

Simultaneously, a blood sample – directly drawn from the main pulmonary artery via a 27 gauge-needle syringe by the surgeon – was used to assess the Svo_2_ before the initiation (T1) and after the termination of CPB (T2). Meanwhile, a blood sample was also taken from the radial artery to measure the arterial blood gas before the initiation (T1) and after the termination of CPB (T2).

It should be mentioned that Blood Pressure (BP), Heart Rate (HR), and Central Venous Pressure (CVP) were continuously monitored and regularly recorded before the induction, immediately and 10 minutes after the induction, and 5, 15, and 30 minutes after the pump termination. In case of uncontrolled hypertension, nitroglycerin (TNG) was used to maintain blood pressure during the surgery. Moreover, in case of hemodynamic instability (mean arterial pressure < 60 mm Hg) at the termination of CPB, epinephrine 0.05 μg / kg / min was started and the infusion rate was increased as needed.

## 2.1 Statistical Analysis

According to power static software collection (SSC), fifty five patients were required for this study to have 80% power to detect significant differences between the corresponding variables (P < 0.05, two-sided).

The study data were transferred into a computer database for further analysis by SPSS for Windows; Version 19.0 (SPSS Inc., Chicago, IL, USA). Paired t-test was used to compare the first and the second sampling within the groups. In addition, Pearson’s correlation was employed to determine the correlation between Svo_2_ and Scvo_2_, Svo_2_ and serum lactate level in the first and second samplings. Moreover, independent t-test was used to compare the demographics characteristics and the coexisting illnesses of the patients. The data were reported as mean ± SD. Besides, two-sided P value less than 0.05 was considered as statistically significant.

## 3. Results

This study was conducted on 62 patients undergoing elective CABG from November 2010 to February 2011 in our center. Demographic characteristics and coexisting illnesses of the patients are presented in Table 1. 

**Table 1.** Demographics Characteristics and Coexisting Illnesses of the Patients[Table-fn fn6751]

Parameter	N = 62	%
**Male / Female**	33 / 29	53.2 / 46.8
**Age of patient (years)**	60.91 ± 1.02	
**Ejection fraction (%)**	48.81 ± 1.07	
**Body mass index (BMI)**	24.48 ± 5.18	
**Hypertension**	33	53.2
**Hypercholesterolemia**	28	44.4
**Diabetes mellitus**	23	36.5
**History of smoking**	15	23.8
**Opium addicts**	10	15.9

Age, ejection fraction, and body mass index data are represented as mean ± standard deviation

**Figure 1.**
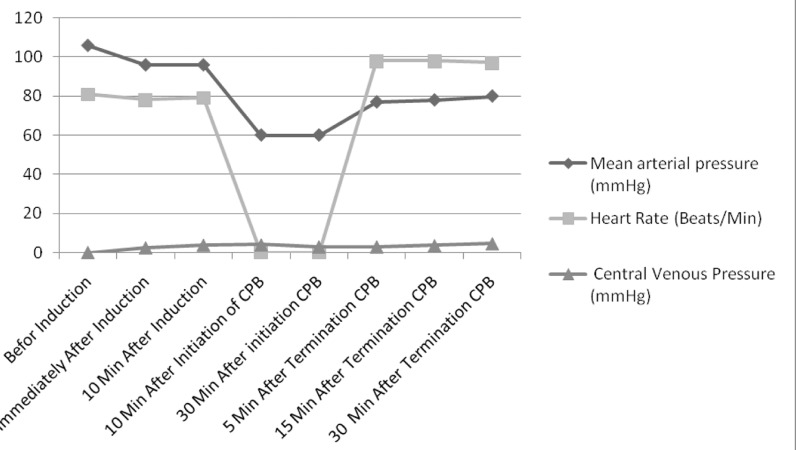
Hemodynamic Parameters During Coronary Artery Bypass Graft Monitoring

Figure 1 summarizes the recorded BP, HR, and CVP during the surgery. Table 2 compares the means of Svo_2_, Scvo_2_, and lactate levels before and after the termination of CPB. The results revealed no significant change in Svo_2_ and radial artery oxygen saturation (P = 0.839 and P = 0.530, respectively). However, statistically significant changes were observed in Scvo_2_ and lactate levels before and after the CPB (P < 0.001).

**Table 2.** Mean Mixed Venous Oxygen Saturation, Central Venous Oxygen Saturation, and Lactate Levels Before and After the Cardiopulmonary Bypass (CPB)

	Before the CPB	After the CPB	*P* value
**Pulmonary artery oxygen saturation (%)**	69.82 ± 10.89	69.90 ± 12.88	0.839
**Radial artery oxygen saturation (%)**	99.19 ± 1.41	99.01 ± 2.23	0.530
**Central venous oxygen saturation (%**)	66.49 ± 13.56	74.93 ± 11.32	< 0.001
**Lactate concentration (mmol / L)**	1.14 ± 0.51	2.71 ± 1.29	< 0.001

**Figure 2.**
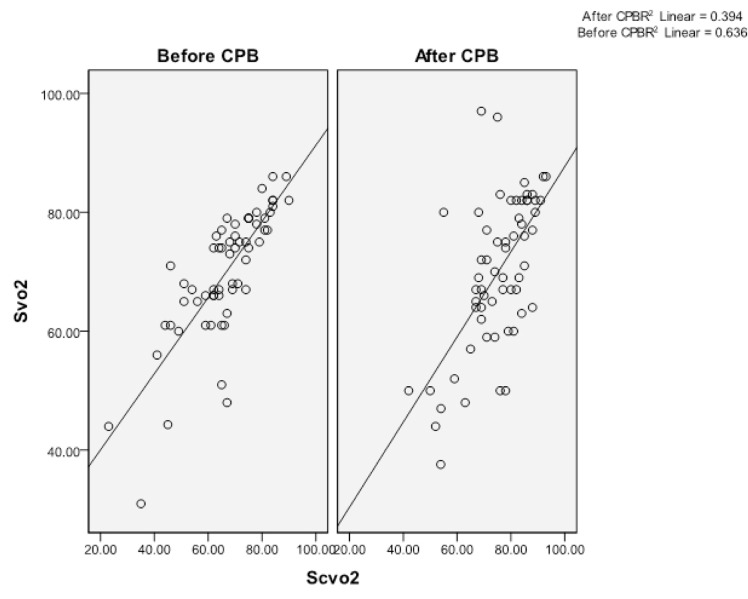
The Correlation Between Central Venous Saturation (Scvo_2_) and Mixed Venous O_2_ Saturation (Svo_2_) Before CPB (r = 0.797, P < 0.001) and After Termination CPB (r = 0.63, P < 0.001).Cardiopulmonary Bypass (CPB)

Table 3 indicates the correlations between Svo_2_ and other markers (Scvo_2_ and lactate level before and after the termination of CPB). The study findings showed a significant correlation between Svo_2_ and Scvo_2_ (P < 0.05) before and after CPB (Figure 2). However, no significant correlation was found between Svo_2_ and serum lactate level (P > 0.05). Also, no correlations were observed between the lactate changes (T1 subtracted from T2) and variations of Svo_2_ (r = 0.017, P = 0.901) and Scvo_2_ (r = 0.127, P = 0.343) before and after the CPB.

**Table 3.** Correlations Between Svo_2_, Scvo_2_, and Serum Lactate Level Before and After the Cardiopulmonary Bypass[Table-fn fn6752]

Before CPB	r-value	*P* value
**Svo2**		
Scvo2	0.797	< 0.001
Serum lactate level	0.137	0.300
After termination CPB	r-value	*P* value
**Svo2**		
Scvo2	0.63	< 0.001
Lactate serum level	0.124	0.348

Abbreviations: CPB, cardiopulmonary bypass; Svo2, Mixed venous oxygen saturation, SCvo2, central venous oxygen saturation

In this study, the mean pump time was 74.67 ± 20.72 minutes with the mean cross clamp time of 41.90 ± 12.49 minutes. The serum lactate level after the CPB was positively correlated with the cross clamp time (r = 0.45, P = 0.001). Lactate variations (T1 subtracted from T2) were also positively correlated with the cross clamp time (r = 0.50, P < 0.001).

## 4. Discussion

Up to now, Svo_2_ has been routinely used as a standard method for assessment of tissue perfusion (1). However, this method is invasive, costly, and can lead to considerable complications during or after the surgery (5). It was shown in major abdominal surgery that Scvo_2_ could reflect important changes in O_2_ delivery in relation to O_2_ requirements during the perioperative period (14). Thus, the present study aimed to evaluate whether Scvo_2_ can be substituted with other markers, such as serum lactate level, during on pump CABG.

The animal studies have reported a correlation between Svo_2_ and Scvo_2_. For instance, Schou et al. (7) found a correlation coefficient of 0.97 in hypoxic pigs. However, Lorentzen et al. (6) compared Svo_2_ and Scvo_2_ in two kinds of cardiac surgeries (aortic valve surgery and CABG) and found that Scvo_2_ might show a trend if cardiac consumption remains constant. Therefore, it could not be a suitable substitute especially in aortic valve surgery and CABG where there is an increase in cardiac O_2_ consumption during the surgery. Similarly, other studies have shown that Scvo_2_ is not as useful as Svo_2_ in critically ill patients, but it can be substituted whenever absolute values are not required (1, 2, 15, 16). On the other hand, the findings of the present study indicated a significant correlation between Svo_2_ and Scvo_2_ before starting the bypass and after the termination of CPB. On the whole, tissue perfusion could be followed by Scvo_2_ during on pump CABG without the need for insertion of PAC and the related complications.

In our study, the serum lactate level was significantly changed during CPB (mean difference between before and after CPB). However, this mean difference and the mean lactate level before and after the termination of CPB showed no correlations with Svo_2_ and Scvo_2_. Therefore, it can be concluded that lactate level is not a reliable substitution for Svo_2_ for assessment of oxygen consumption during cardiac surgery. Mustafa et al. (5) reported an increase in the serum lactate level after CPB mainly because of a decrease in liver capacity for clearance of lactate level or less possibly because of the higher production of lactate due to inadequacies of oxygen level and glycolysis induction. Nonetheless, Rao et al. (17) indicated that myocardial lactate release during reperfusion was a marker of inadequate myocardial protection during cardioplegic arrest. In the present study, the lactate level and its changes before and after the cardiopulmonary bypass was not parallel to Svo_2_ and Scvo_2_ therefore it was not to be a reliable method for assessment of tissue perfusion during on pump CABG.

Moreover, no significant change was found in Svo_2_ before and after the termination of CPB in the present study; therefore, tissue perfusion was effective. In addition, Scvo_2_ was increased after the termination CBP which also implies the effectiveness of tissue perfusion. However, this change in Scvo_2_ was not exactly parallel to Svo_2_ because of different positions of PAC and CVC. On the other hand, an increase was observed in the lactate level after the termination of CBP due to the decrease in the liver capacity to remove the serum lactate. This was not due to inadequate tissue perfusion because Svo_2_ as a gold standard of tissue perfusion marker showed no significant changes after the termination CPB.

In summary, Scvo_2_ was correlated to Svo_2_ during on pump CABG; thus, Scvo_2_ is considered as the best substitute of Svo_2_ for detecting tissue hypoperfusion during on pump CABG. On the other hand, no significant correlation was found between the serum lactate level and Svo_2_ during on pump CABG. Hence, serum lactate is not a good marker of tissue perfusion and ischemia especially after CPB termination during on pump CABG.
